# A Diagnostic Analysis Workflow to Optimal Multiple Tumor Markers to Predict the Nonmetastatic Breast Cancer from Breast Lumps

**DOI:** 10.1155/2021/5579373

**Published:** 2021-07-08

**Authors:** Nan Jiang, Tian Tian, Xianyang Chen, Guofen Zhang, Lijie Pan, Chengping Yan, Guoshan Yang, Lili Wang, Xuchen Cao, Xin Wang

**Affiliations:** ^1^The First Department of Breast Cancer, Tianjin Medical University Cancer Institute and Hospital, National Clinical Research Center for Cancer, Tianjin 300060, China; ^2^Key Laboratory of Cancer Prevention and Therapy, Tianjin 300060, China; ^3^Tianjin's Clinical Research Center for Cancer, Tianjin 300060, China; ^4^Key Laboratory of Breast Cancer Prevention and Therapy, Tianjin Medical University, Ministry of Education, Tianjin 300060, China; ^5^Department of General Surgery, First Hospital of Tsinghua University, Beijing 100016, China; ^6^Department of General Surgery, Beijing Luhe Hospital, Capital Medical University, Beijing 101149, China; ^7^Bao Feng Biotech (Beijing) Co., Ltd., Beijing, China; ^8^ZhongYuan BoRui Biotech (Zhuhai Hengqin) Co., Ltd., Zhuhai, China; ^9^Department of Laboratory Medicine, First Hospital of Tsinghua University, Beijing 100016, China

## Abstract

**Objective:**

To assess the diagnostic performance of clinically common single markers and combinations to distinguish nonmetastatic breast cancer and benign breast tumor. A predictive model with a better diagnostic ability for nonmetastatic breast cancer was established by using the diagnostic process.

**Methods:**

A total of 222 patients with nonmetastatic breast cancer and 265 patients with benign breast disease were enrolled in this study. CEA, Ca 15-3, Ca 125, Ca 72-4, CYFRA 21-1, FERR, AFP, and NSE were measured by an electrochemiluminescent immunoenzymometric assay on the Elecsys system. There are four key steps for our diagnostic workflow, that is, feature selection, algorithm selection, parameter optimization, and outer test data was used to validate the optimal algorithm and markers.

**Results:**

CEA, Ca 15-3, CYFRA 21-1, AFP, and FERR were selected using the *t*-test in our inner development set. The optimal algorithm among logical regression, decision tree, support vector machine, random forest, and gradient boost machine was selected by 10-fold cross-validation, and we found that random forest and logistic regression are the better classification. The outer test data was used to validate the best markers and classification. The random forest with CEA, Ca 15-3, CYFRA 21-1, AFP, and FERR showed the optimal combination for distinguishing breast cancer and benign breast disease. The AUC value was 0.888, the cut-off point was 0.484, and sensitivity and specificity were 78.9% and 90.1%.

**Conclusions:**

No single marker of these eight markers was good at identifying nonmetastatic breast cancer from benign tumors. But a diagnostic analysis workflow was established to develop a predictive model with better diagnostic capability for nonmetastatic breast cancer. This workflow is also applicable to the optimization of other disease markers and diagnostic models. The predictive model showed good diagnostic performance, and it could be gradually incorporated as a support method for the diagnosis of nonmetastatic breast cancer.

## 1. Introduction

Breast cancer is by far the most frequently diagnosed cancer among women with an incidence of 11.6% and overall cancer mortality of 6.6% worldwide [1]. There were an estimated 2.0 million new cases (24.2% of all cancers in women) and 0.6 million cancer deaths (15.0% of all cancer deaths in women) in 2018 [1]. Early diagnosis plays an important role in optimizing treatments and reducing the mortality of breast cancer patients [2]. Screening of early breast cancer forms part of the state programme of routine annual or biannual ultrasonography or mammograms for women within a certain age range [3], in China, between 40 and 70 years old. At present, mammography is the most common screening method for the detection of breast cancer. However, the results are not particularly satisfactory because of the high false-positive and false-negative rates [4]. Ultrasonography is also used for the early diagnosis of breast cancer in China. Unfortunately, approx. 20% of breast cancer patients cannot be diagnosed [5]. Therefore, a complementary instrument is required to get better results for the early diagnosis of breast cancer.

The common tumor markers in clinical use are dominant for various tumors, such as carcinoembryonic antigen (CEA) for colorectal cancer, alpha-fetoprotein (AFP) for hepatocellular carcinoma, Ca 12-5 for ovarian cancer, and so forth [6]. But they cannot be used as effective indicators for the diagnosis of breast cancer. There is no clinical guide, or even consensus among experts, with regard to the use of biomarkers for the early diagnosis of breast cancer. CEA and Ca 15-3 are recommended only for therapeutic monitoring of breast cancer and early detection of recurrent disease but not for breast cancer detection because of their low sensitivity [7–9]. However, many cancers may not be detected by their dominant markers but by the elevation of tumor markers not recommended for monitoring their tumor activity [10]. And screening with multiple tumor markers also allows cancers to be detected in the absence of their dominant markers [10].

Multivariate statistics combined with machine learning as a means of clinical data analysis have been reported in many pieces of literature, especially in the field of breast imaging [11]. However, for the study of molecular markers distinguishing benign and malignant breast diseases, there is only the selection of potential universal markers that have been made and no report on the optimization of indicators and classifiers. In this study, we compared the levels of marker panel (CEA, Ca 15–3, Ca 125, Ca 72-4, cytokeratin fragment 19 (CYFRA 21-1), ferritin (FERR), AFP, and neuron-specific enolase (NSE)) in breast cancer with that in benign controls, respectively, and tried to find an effective marker combination and a better diagnostic capability for nonmetastatic breast cancer.

## 2. Materials and Methods

### 2.1. Patients

The development set included 111 breast cancer and 132 benign samples, which were obtained from the First Hospital of Tsinghua University. The study was performed according to the standards of the Institutional Ethical Committee and the Helsinki Declaration and was approved by the clinical ethics committee of the Tsinghua University. The patients with breast cancer were selected according to the following criteria: (1) all patients with breast cancer were diagnosed by pathology; (2) all patients were female; (3) all patients had no distant metastases; (4) no patients received prior neoadjuvant oncological treatment; and (5) no patients were previously diagnosed with any other tumor. The patients with benign breast diseases were selected according to the following criteria: (1) all patients with benign breast diseases were diagnosed by pathology; (2) all patients were female; (3) no patients were previously diagnosed with any other tumor.

According to these criteria, we collected a validation set, including 111 breast cancer and 133 benign samples from the First Hospital of Tsinghua University, as the outer test group. All of these cancer and benign samples were approximately age-matched and were included according to the above criteria. All patients underwent surgical resection of the tumors. The clinicopathological characteristics and tumor stage were assessed based on the histopathological results.

### 2.2. Marker Analysis

Tumor marker measurements were performed strictly according to the manufacturer's instructions and quality control was ensured. Before surgery and after overnight fasting, 10 ml of venous blood was collected into a vessel tube containing heparin as an anticoagulant from each subject and was subsequently centrifuged (1500 × g for 15 min) to collect clear serum. The sera were then transferred into sterile vials and immediately stored at −80°C until further analysis. Subsequently, CEA, Ca 15-3, Ca 125, Ca 72-4, CYFRA 21-1, FERR, AFP, and NSE were assessed by an electrochemiluminescent immunoenzymometric assay (Roche Diagnostics, Germany) on the Elecsys system.

### 2.3. Marker and Model Optimization

**Figure 1:**
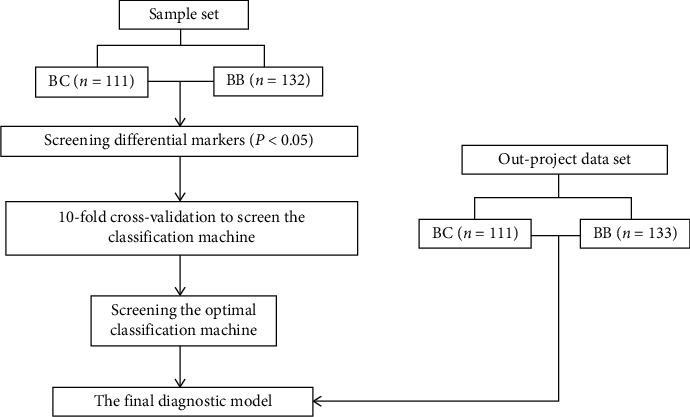
A workflow to develop a better diagnostic capability for nonmetastatic breast cancer. BC means breast cancer patients; BB means benign breast disease patients.

We aimed to build a binary classifier that can distinguish between the nonmetastatic breast cancer and benign breast tumors accurately. The workflow is described in Figure 1.

There are four key steps for our diagnostic workflow, that is, feature selection, algorithm selection, parameter optimization, and an outer validation for the optimal algorithm and markers. All the analysis was performed using rpart, random forest, e1071, gbm packages of *R* software (http://www.r-project.org).

In the main modeling and mining methods, the “glm” function in the “stat” package is used for logistic regression, the family parameter is set as “logit”, and the default parameters are used for the rest. The decision tree uses the “rpart” function in the “rpart” package, sets the method parameter as “class”, and uses the default parameters for the rest. Random forest uses the “randomForest” function in the randomForest package and sets the “mtry” parameter as “2”, the “ntree” parameter as “500”, the proximity parameter as “*T*”, and the importance parameter as “*T*”. The SVM model uses the “svm” function in the “e1071” package, and the default parameters in the function are used for the parameters.

Student's *t*-test was carried out using internal training data to obtain the different indicators as potential markers between breast cancer and benign breast diseases.

The 10-fold cross-validation was carried to ensure the repeatability of the results by setting random seeds. By evaluating the performance of the training model, the optimal algorithm is selected in logistic regression, decision tree, support vector machine, random forest, and gradient boost machine. The sensitivity, specificity, accuracy, and area under the curve (AUC value) were determined. All the values are calculated as the mean value based on the inner training data was randomly divided into 10 subsets with equal sizes, and a single subset is retained as the validation data for evaluating the model, and the remaining 9 subsets are used for training.

Finally, the outer test data is used as external validation to verify the optimization algorithm and tags.

### 2.4. Statistical Analysis

Results are expressed as the mean ± SD for continuous variables and as the number (percent) for categorical variables. All statistical analyses were conducted using *R* software version 2.9.1. All statistical analyses were carried out using *R* software version 2.9.1. The differences of tumor markers between breast cancer and benign breast diseases were compared. When the data obeyed normal distribution, *t*-test was used; otherwise, Wilcoxon rank-sum test was used.

## 3. Results

### 3.1. Patient Characteristics

A total of 222 patients with nonmetastatic breast cancer and 265 patients with benign breast disease were enrolled in our study. All subjects were female from Han Chinese. The basic clinical and biological characteristics of the nonmetastatic breast cancer patients in the development set and validation set enrolled in this study are summarized in Table 1. The mean age of patients with benign breast disease was 42.6 ± 12.6 years in the development set and 42.7 ± 12.4 years in the validation set. There is no difference in clinicopathological characteristics between the two groups.

### 3.2. Blood Biomarkers Analysis

**Figure 2:**
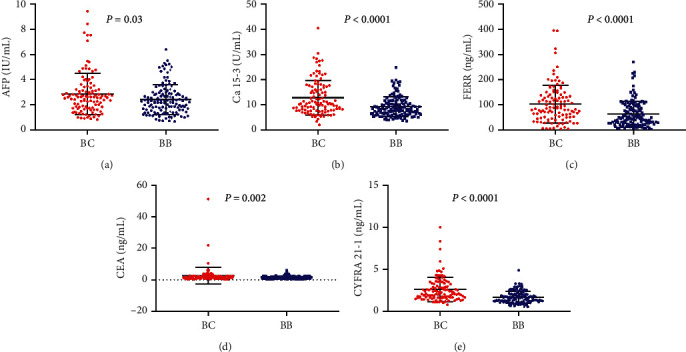
The expression levels of five differentiating tumor biomarkers between breast cancer patients and benign breast disease patients. The start means a significant difference between breast cancer patients compared to benign breast disease patients.

The levels of serum CEA, Ca 15-3, Ca 125, Ca 72-4, CYFRA 21-1, FERR, AFP, and NSE in all patients were analyzed. Univariate statistical analysis using the *R* project was performed to validate the statistical significance (*P* < 0.05) of the tumor biomarker differences between breast cancer patients and benign breast disease patients. Five tumor biomarkers were selected with *P* < 0.05 (Table 2). These five differentiating tumor biomarkers, including CEA, Ca 15-3, CYFRA 21-1, AFP, and FERR showed increased levels in breast cancer patients compared with benign breast disease patients (Figure 2).

### 3.3. Relationships between Serum Biomarkers and Clinical Characteristics of Breast Cancer Patients

**Figure 3:**
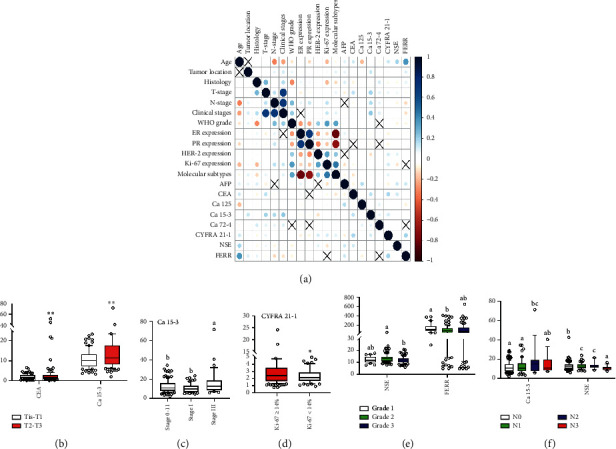
(a): Red represents negative correlation, and blue represents a positive correlation. The color depth represents the degree of correlation: a deeper color indicates a higher correlation. (b) Levels of CEA and Ca 15-3 in Tis-T1 versus T2-3 (*P* < 0.05). (c) Level of Ca 15-3 in different clinical stages (*P* < 0.05). (d) Level of CYFRA 21-1 in patients with the Ki-67 ≧ 14% versus Ki-67 < 14% (*P* < 0.05). (e) Levels of NSE and FERR in different tumor grades. (f) Levels of Ca 15-3 and NSE in different *N*-stage (*P* < 0.05).

The levels of these eight tumor biomarkers in 222 breast cancer patients of the development and validation set with different clinicopathological characteristics were analyzed to investigate the relationship between these eight tumor biomarkers and the clinical characteristics of the patients. We performed a matrix correlation analysis of tumor biomarkers and clinicopathological characteristics of patients with breast cancer, which can be seen from the graph (Figure 3(a)). The changes in the levels of these eight biomarkers were not correlated with histology and molecular subtypes. However, the significant difference between CEA and Ca 15-3 levels was higher in Tis-T1 than in T2-3 (*P* < 0.05) (Figure 3(b)). And for the clinical staging of breast cancer, Ca 15–3 levels were also higher in stage III than that in stage I and stages 0-II, respectively (*P* < 0.05) (Figure 3(c)). For the molecular marker, CYFRA 21-1 had higher levels in patients with the expression of Ki-67 at ≥14% (*P* < 0.05) (Figure 3(d)). Furthermore, NSE was also downregulated in grade III patients compared with grade II patients (*P* < 0.05); and FERR was downregulated in grade II patients compared with grade I patients (*P* < 0.05) (Figure 3(e)). Considering the staging of lymph nodes, the result showed that Ca 15-3 was upregulated in N2 patients compared with N0 and N1 patients, respectively (*P* < 0.05) (Figure 3(f)). But, NSE was downregulated in N3 patients compared with N0, N1, and N2 patients, respectively (*P* < 0.05).

### 3.4. Differential Diagnostic Value of Biomarkers

**Figure 4:**
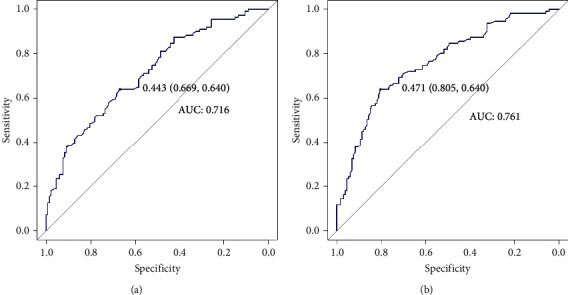
ROC analyses of CEA (a) and CYFRA 21-1 (b) to distinguish breast cancer patients from benign breast disease patients.

The capacity of these five tumor markers to differentiate breast cancer patients from patients with benign breast disease was assessed with ROC analysis. CEA (AUC 0.716) and CYFRA 21-1 (AUC 0.761) showed good diagnostic performance. Sensitivity and specificity are 64.0% and 66.9% for CEA, and 64.0% and 80.5% for CYFRA 21-1 (Figure 4; Table 3).

### 3.5. Establishment and Validation of a Predictive Model

Multivariate statistical analysis was used for further research. We chose logistic regression, decision tree, random forest, support vector machine, and gradient boost machine as alternative algorithms. Through the 10-fold cross-validation, the metrics of each model were calculated, respectively, including accuracy, sensitivity, specificity, and AUC. According to the statistical analyses of the results of 10 verifications, we found that logistic regression had a similar classification effect with random forest, which was specifically shown as high AUC value and accuracy (Table 4).

**Figure 5:**
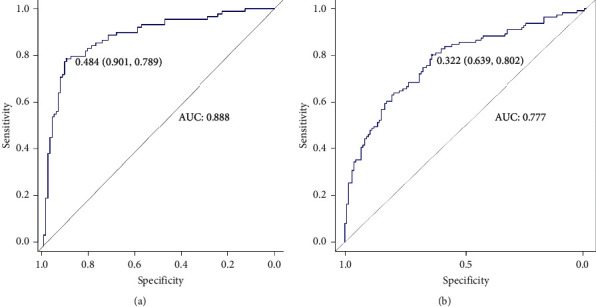
ROC analyses of the models of random forest (a) and logical regression (b) through external validation to distinguish breast cancer patients from benign breast disease patients.

**Figure 6:**
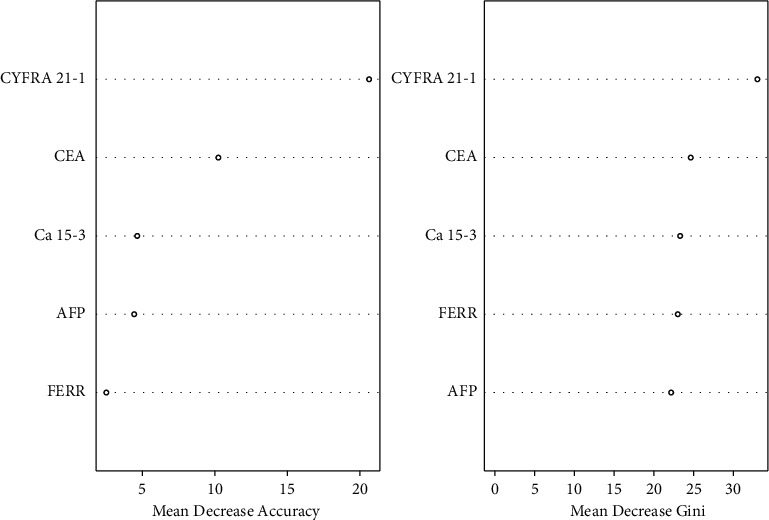
The importance of variables in the random forest model.

Later, we used outer validation data to perform the out-of-project test. Through ROC comparison, we found that random forest showed the best diagnostic performance with AUC of 0.888, sensitivity of 78.9%, and specificity of 90.1% (Figure 5(a)), compared to the AUC of 0.777 in the logical regression model (Figure 5(b)). Also, variables importance in the model of the random forest was analyzed to evaluate the importance of variables from two perspectives: Mean Decrease Accuracy and Mean Decrease Gini. The results showed that CYFRA 21-1, CEA, and Ca 15-3 were the three most important variables in the model (Figure 6).

## 4. Discussion

In the present analysis, we investigated a panel of different markers to define which marker or which combination can be used in detecting nonmetastatic breast cancer from breast lumps and to develop a workflow with better diagnostic capability for nonmetastatic breast cancer.

A total of eight clinically used markers, including CEA, Ca 15-3, Ca 125, Ca 72-4, CYFRA 21-1, FERR, AFP, and NSE, were detected in all patients. Among them, five markers such as CEA, Ca 15-3, CYFRA 21-1, FERR, and AFP were found to have important differences between breast cancer and benign tumors.

Fold change value was calculated by the average value of breast cancer divided by the average value of benign breast disease. Fold change with a value larger than 1 indicates a higher level of the biomarker in plasma of breast cancer, while a fold change value lower than 1 indicates a lower level, compared to benign breast disease.

In the present analysis, Ca 15-3, FERR, and AFP showed increased levels in breast cancer patients compared with the benign breast disease controls. Ca 15-3, a variant of mammary epithelial surface glycoprotein and an antigen related to breast cancer, are used for therapeutic monitoring of breast cancer and early detection of recurrent disease [6–8]. Choi et al. [12] found that the levels of Ca 15-3 were higher in breast cancer patients than in benign breast disease by an antibody-lectin Sandwich assay that appeared to efficiently discriminate nonmetastatic breast cancer from benign breast disease. In our study, the Ca 15-3 level was also upregulated in breast cancer patients. However, Ca 15-3 showed a poor diagnostic ability, which might be caused by different detection methods. Also, the serum level of Ca 15-3 was associated with host tumor burden such as larger tumor size, more lymph node metastases, and advanced stage. Therefore, preoperative high serum levels of Ca 15-3 may indicate a poor outcome.

Ferritin is currently used to monitor the presence of malignant disease; it is regarded as a predictor of positive lymph nodes involved in patients with breast cancer [13, 14]. Orlandi et al. [14] found that breast cancer patients had significantly higher ferritin levels compared with the benign breast disease controls, which is consistent with our research. Several studies indicate that plasmatic ferritin is produced and secreted by macrophages, hepatocytes, and cancer cells [15], which may be the reason for the high levels of ferritin in breast cancer.

AFP, used as a liver cancer biomarker for over 30 years, may also be elevated to varying degrees in patients with gastric cancer, pancreatic cancer, or lung cancer [16–19]. He et al. [20] found that the median value of AFP in 17 kinds of diseases was higher than that in healthy controls, including breast cancer. Little literature has studied the difference in AFP levels between benign and malignant breast cancer. Our results showed that AFP was elevated in breast cancer patients compared with the benign breast disease controls. The above summary indicated that both the source and the regulation of serum AFP levels were much more complicated than previously thought.

CEA and CYFRA 21-1 also showed increased levels in breast cancer patients compared with the benign breast disease controls. Concerning the differentiation between the two groups, CEA (AUC 0.716) and CYFRA 21-1 (AUC 0.761) showed good diagnostic performance. Also, CEA and CYFRA 21-1 were directly associated with larger tumor size and high Ki-67 index, respectively, in our study. Since tumor size and Ki-67 level were positively correlated with host tumor burden and malignant degree of breast cancer, respectively [21, 22], preoperative elevated levels of serum CEA and CYFRA 21-1 could be related to a poor outcome. CEA, a widely used tumor marker for examination and prediction in many cancers [23] and CYFRA 21-1, an excellent tumor marker in lung cancer [24, 25], were found upregulated in breast cancer patients in several studies [26–30]. They are consistent with our findings. However, when we performed cross-validation within the test group, we found that the AUC values of CEA and CYFRA 21-1 were not stable. In conclusion, this means that no single marker of these five markers is well-diagnosed for breast cancer.

One relevant finding of the present work has been the design of a final predictive model. Some reports used the combination of molecular markers to identify breast cancer [26, 30, 31]. Bayo et al. developed a predictive model using NSE, Ca 15-3, NGAL, EGFR, and 8-OHdG for early breast cancer diagnosis with AUC of 0.918 [26]. Liu et al. used a panel of PD-1, IL-10, IL-2R*α*, and Ca 15-3 for early-stage breast cancer diagnosis; this panel also had the AUC of 0.811 [31]. They had used newly discovered molecular markers in combination with classic tumor markers to improve the diagnosis rate of breast cancer. However, the diagnostic value had not been verified, and the new molecular markers have a longer turnaround time and many uncertainties from discovery to clinical application. In our study, the markers we selected were all tumor markers commonly used in clinical practice, and the model we established still had a stable and good diagnostic effect after validation. Since these markers have been widely used in various medical institutions, this diagnostic tool would be easily promoted and applied.

Some literature [8, 32] reported that simultaneous use of CEA and Ca 15-3 allowed the early diagnosis of metastasis in up to 60–80% of patients with breast cancer. Moreover, CEA and Ca 15-3 have been shown to detect 40–60% of breast cancer recurrences before clinical or radiological evidence of disease. Our study only contains data on early breast cancer, and some patients with advanced breast cancer should be added in the future. In addition, CEA and Ca 15-3 have been able to predict the recurrence of breast cancer [33]. I believe that the combined application of these five markers can better predict the recurrence of breast cancer, which will also be the direction of our future work.

At the beginning of the modeling, it was found that the AUC mean of the logistic regression model was similar to the random model in the internal 10-fold cross-validation. The result indicated that the linear generalized regression method might have a similar ability of internal stability control as the nonparametric probabilistic method. However, in the external verification, the random forest algorithm performs better and stronger external generalization ability, which is more in line with clinical application. The relationship between outcome variables and multiple indicators often cannot be parameterized by simple linear means. The same is true for the markers of breast cancer in our study, while the projection of variables to the high-dimensional space in the modeling is not completely linear. Therefore, the nonparametric method can be better fitted for the relationship between outcome variables and multiple indicators, and the importance of variables in the random forest model was also more reliable (Figure 5). In our future studies, a larger population to obtain more general data results is required to prove that the workflow is widely applicable and robust in the different cohorts.

Our research had the following limitations. First, our study population only consisted of Chinese women patients with breast cancer. In future studies, the scope could be broadened to include other ethnicities. Second, although the sample size was relatively large, the study population was selected from one hospital, and more validation would be carried out in other research institutions.

## 5. Conclusions

In summary, CEA, Ca 15-3, CYFRA 21-1, FERR, and AFP were found to be elevated in nonmetastatic breast cancer patients compared with the benign breast disease controls in our study. However, no single marker of these five markers is good at identifying breast cancer from benign tumors. A diagnostic analysis workflow was established to develop a better diagnostic capability for nonmetastatic breast cancer. This workflow is also applicable to the optimization of other disease markers and diagnostic models. The predictive model showed good diagnostic performance with AUC of 0.888, sensitivity of 78.9%, and specificity of 90.1%, and it could be gradually incorporated as a support method for the diagnosis of nonmetastatic breast cancer.

## Figures and Tables

**Table 1:** Comparison of clinicopathological characteristics in patients with nonmetastatic breast cancer in development set and validation set.

Characteristic	Number of patients (%)		*P* value
	Development set	Validation set	
Age (years)			
Mean ± SD	57.5 ± 12.9	57.1 ± 14.6	0.829
Range	29–86	22–91	

Tumor location			0.591
Left	55 (49.5%)	51 (49.5%)	
Right	56 (50.5%)	60 (50.5%)	

Histology			0.868
In situ	5 (4.5%)	6 (5.4%)	
Ductal	77 (69.4%)	82 (73.9%)	
Lobular	4 (3.6%)	2 (1.8%)	
Others	25 (22.5%)	21 (18.9%)	

T-stage			0.391
Tis	5 (4.5%)	6 (5.4%)	
T1	39 (35.1%)	53 (47.8%)	
T2	62 (55.9%)	48 (43.2%)	
T3	5 (4.5%)	4 (3.6%)	

N-stage			0.824
N0	74 (66.7%)	66 (59.5%)	
N1	25 (22.5%)	28 (25.2%)	
N2	6 (5.4%)	9 (8.1%)	
N3	6 (5.4%)	8 (7.2%)	

WHO grade			0.664
I	13 (11.7%)	17 (15.3%)	
II	50 (45.1%)	51 (46.0%)	
III	48 (43.2%)	43 (38.7%)	

Clinical stages			0.584
0	5 (4.5%)	6 (5.4%)	
I	28(25.2%)	33(29.7%)	
II	65 (58.6%)	53 (47.8%)	
III	13 (11.7%)	19 (17.1%)	

ER expression			0.613
Positive	87 (78.4%)	81 (73.0%)	
Negative	20 (18.0%)	24 (21.6%)	
N/A	4 (3.6%)	6 (5.4%)	

PR expression			0.763
Positive	72 (64.9%)	73 (65.8%)	
Negative	35 (31.5%)	32 (28.8%)	
N/A	4 (3.6%)	6 (5.4%)	

HER-2 expression			0.638
Positive	29 (26.1%)	33 (29.7%)	
Negative	78 (70.3%)	72 (64.9%)	
N/A	4 (3.6%)	6 (5.4%)	

Ki-67 expression			0.291
<14%	28 (25.2%)	37 (33.3%)	
≥14%	79 (71.2%)	68 (61.3%)	
N/A	4 (3.6%)	6 (5.4%)	

Molecular subtypes			0.203
Luminal A	20 (18.0%)	30 (27.0%)	
Luminal B	68 (61.3%)	52 (46.9%)	
HER-2 (+)	8 (7.2%)	14 (12.6%)	
Basal-like	11 (9.9%)	9 (8.1%)	
N/A	4 (3.6%)	6 (5.4%)	

ER: estrogen receptor; PR: progesterone receptor; HER-2: human epidermal growth factor receptor 2; N/A: not available.

**Table 2:** Comparison of plasma biomarkers levels in breast cancer patients and benign breast disease patients.

Markers	Mean ± SD		*P* value	Fold change
	Breast cancer	Benign breast disease		
CEA (ng/mL)	2.58 ± 5.24	1.47 ± 0.87	0.002	1.76
Ca 15-3 (U/mL)	12.84 ± 6.9	9.2 ± 3.98	0.000	1.4
CYFRA 21-1 (ng/mL)	2.59 ± 1.46	1.66 ± 0.71	0.000	1.55
AFP (IU/mL)	2.86 ± 1.64	2.41 ± 1.18	0.030	1.19
FERR (ng/mL)	102.81 ± 75.45	63.2 ± 51.52	0.000	1.63
Ca 12-5 (U/mL)	14.39 ± 10.97	15.17 ± 8.66	0.192	0.95
Ca 72-4 (U/mL)	3.8 ± 4.07	3.15 ± 3.39	0.237	1.21
NSE (ng/mL)	12.21 ± 2.95	12.05 ± 2.82	0.667	1.01

*P* values are calculated from the Wilcoxon rank-sum test.

**Table 3:** Diagnostic performance of serum biomarkers in discriminating breast cancer from benign breast diseases.

Markers	Cut-off point	AUC	Sensitivity	Specificity
CEA	0.443	0.716	0.64	0.669
Ca 15-3	0.5	0.648	0.387	0.85
CYFRA 21-1	0.471	0.761	0.64	0.805
AFP	0.433	0.577	0.604	0.549
FERR	0.647	0.592	0.27	0.902

**Table 4:** Calculation of accuracy, sensitivity, specificity, and AUC after 10-fold cross-validation for different classifiers.

	Accuracy (%)	Sensitivity	Specificity	AUC
Logistic regression	71.316	0.713	0.632	0.789
Decision tree	65.466	0.683	0.619	0.703
Support vector machine	70.9	0.697	0.597	0.684
Random forest	71.699	0.721	0.666	0.772
Gradient boosting machine	68.4	0.68	0.614	0.679

## Data Availability

The datasets used and/or analyzed during the current study are available from the corresponding author on reasonable request.
